# Implementation and Evaluation of Resuscitation Training for Childcare Workers

**DOI:** 10.3389/fped.2022.824673

**Published:** 2022-02-28

**Authors:** Jörg Michel, Tim Ilg, Felix Neunhoeffer, Michael Hofbeck, Ellen Heimberg

**Affiliations:** Department of Pediatric Cardiology, Pulmonology and Pediatric Intensive Care Medicine, University Children’s Hospital Tübingen, Tübingen, Germany

**Keywords:** basic life support, resuscitation, pediatric critical care, kindergarten, daycare, pediatric out-of-hospital cardiac arrest, emergency

## Abstract

**Background and Objective:**

Children spend a large amount of time in daycare centers or schools. Therefore, it makes sense to train caregivers well in first-aid measures in children. The aim of this study is to evaluate whether a multimodal resuscitation training for childcare workers can teach adherence to resuscitation guidelines in a sustainable way.

**Materials and Methods:**

Caregivers at a daycare center who had previously completed a first-aid course received a newly developed multimodal resuscitation training in small groups of 7–8 participants by 3 AHA certified PALS instructors and providers. The 4-h focused retraining consisted of a theoretical component, expert modeling, resuscitation exercises on pediatric manikins (Laerdal Resusci Baby QCPR), and simulated emergency scenarios. Adherence to resuscitation guidelines was compared before retraining, immediately after training, and after 6 months. This included evaluation of chest compressions per round, chest compression rate, compression depth, full chest recoil, no-flow time, and success of rescue breaths. For better comparability and interpretation of the results, the parameters were evaluated both separately and summarized in a resuscitation score reflecting the overall adherence to the guidelines.

**Results:**

A total of 101 simulated cardiopulmonary resuscitations were evaluated in 39 participants. In comparison to pre-retraining, chest compressions per round (15.0 [10.0–29.0] vs. 30.0 [30.0–30.0], *p* < 0.001), chest compression rate (100.0 [75.0–120.0] vs. 112.5 [105–120.0], *p* < 0.001), correct compression depth (6.7% [0.0–100.0] vs. 100.0% [100.0–100.0], *p* < 0.001), no-flow time (7.0 s. [5.0–9.0] vs. 4.0 s. [3.0–5.0], *p* < 0.001), success of rescue breaths (0.0% [0.0–0.0] vs. 100.0% [100.0–100.0], *p* < 0.001), and resuscitation score were significantly improved immediately after training (3.9 [3.2–4.9] vs. 6.3 [5.6–6.7], *p* < 0.001). At follow-up, there was no significant change in chest compression rate and success of rescue breaths. Chest compressions per round (30.0 [15.0–30.0], *p* < 0.001), no-flow time (5.0 s. [4.0–8.0], *p* < 0.001), compression depths (100.0% [96.7–100.0], *p* < 0.001), and resuscitation score worsened again after 6 months (5.7 [4.7–6.4], *p* = 0.03). However, the results were still significantly better compared to pre-retraining.

**Conclusion:**

Our multimodal cardiopulmonary resuscitation training program for caregivers is effective to increase the resuscitation performance immediately after training. Although the effect diminishes after 6 months, adherence to resuscitation guidelines was significantly better than before retraining.

## Introduction

Although out-of-hospital cardiac arrest (OHCA) is much less common in children than in adults, it is a serious event that can theoretically occur anywhere and at any time both in chronically ill and in healthy children (1, 2). Survival after OHCA is poor and long-term sequelae are common. Immediate performance of bystander cardiopulmonary resuscitation (CPR) is important for improving outcome but is often not performed (3–5). Due to the rare nature of pediatric OHCA and the immensely stressful and distressing situation for those present, training is recommended for all potentially involved persons such as children, parents, childcare workers, and school personnel (6). Although this effort would be highly desirable, focused resuscitation training to all potential first responders is time consuming and costly. Since children spend a large part of their time in daycare centers or schools and the number of caregivers is relatively small, it is a logical approach to offer them professional training in first-aid measures in children. In 2019, the American Academy of Pediatrics, the American Public Health Association, and the National Resource Center for Health and Safety in Child Care and Early Education released the 4th edition of “Caring for Our Children: National Health and Safety Performance Standards; Guidelines for Early Care and Education Programs” (7). It recommends that at least one staff member qualified to respond to life-threatening emergencies must be present at all times. The staff member is required to be competent in first aid, pediatric CPR, and management of a blocked airway. Retraining is recommended every 2–3 years, depending on the course provider. Video-based self-learning kits from the AHA are mentioned as a cost-effective alternative. A survey of childcare centers in Pennsylvania found that 77% of centers require mandatory CPR training even for all staff. Yet only 55% of respondents answered that they felt “prepared” or “very prepared” to respond to urgent medical situations (8). In Germany, where the study was conducted, one staff member trained in first aid is mandatory for each group in daycare centers. Retraining must be carried out every 2 years. There is no specification of the duration, content, and scope of the training courses. The maximum number of participants is limited to 20 persons per instructor (9).

We therefore developed a standardized multimodal resuscitation program for childcare workers at daycare centers. The aim of this study is to verify whether our newly developed resuscitation training program can teach adherence to resuscitation guidelines in a sustainable way. In addition, we compare the resuscitation skills immediately after retraining and after a period of about 6 months with the situation before retraining.

## Materials and Methods

Childcare workers at a daycare center who had previously completed at least one conventional BLS training received a new multimodal cardiopulmonary resuscitation retraining on a voluntary basis. The previous BLS training was a first aid course in a large group of up to 20 participants per 1 instructor with a duration of about 4 h. In the first aid courses that are usually conducted in Germany, the BLS measures are often well explained. However, simulated scenarios are not performed (10).

The study protocol was approved by the local Ethics Committee of the University Hospital Tübingen (258/2019BO2).

### Pre-retraining Assessment

To assess the pre-retraining situation, participants were asked to perform single-rescuer CPR with an infant manikin for 2 min (Laerdal Resusci Baby QCPR; Laerdal Medical AS, Stavanger, Norway) prior to the retraining. The performed steps were documented (check for responsiveness, shout for nearby help, look for breathing, activate emergency response system) and analyzed using a laptop with a software which reported quality of chest compressions and ventilations (Laerdal Wireless SkillReporting; Laerdal Medical AS, Stavanger, Norway). In addition, we gave participants a mini-survey consisting of the following items: are you confident to perform resuscitation on a child? If no, why not? Does the thought of potential emergencies at work make you anxious? Do you think that the emergency training courses you have attended so far have prepared you well for potential emergency situations?

### Emergency and Cardiopulmonary Resuscitation Training Program

The training program was designed for 7–8 participants with a duration of 4 h. Training was delivered by two American Heart Association (AHA) Pediatric Advanced Life Support (PALS) instructors and one AHA PALS provider. The first part of the training consisted of a theoretical session on possible pediatric emergencies such as unconsciousness, choke, drowning, or injuries, and their prevention and first aid measures. In the practical part, the focus was on retraining CPR measures. Instructors first demonstrated with the manikins how to perform single-rescuer and two or more rescuer pediatric basic life support (BLS) according to the 2015 guidelines of the AHA (2, 11). Although the training was performed in Germany/Europe and there are resuscitation guidelines from the European Resuscitation Council (ERC) (12), this study was performed based on the AHA guidelines. This is because our university children’s hospital is a certified AHA training center and all trainers are certified AHA PALS instructors. In addition, the two guidelines are similar in most aspects, and we sought to allow replication of the training concept and study outside of Europe. Because there was no automated external defibrillator (AED) at the facility, the use of an AED was not a part of this study. During the BLS training, the important part of the AED in the guidelines and the usefulness and immense importance of an AED for the therapy of cardiac arrhythmias as the cause of cardiac arrest were highlighted. Following the CPR demonstration, all participants practiced single-rescuer resuscitation according the BLS guidelines. To open the airway and to perform mouth-to-mouth or mouth-to-nose ventilation (MMV), participants were instructed to perform head-tilt chin-lift and jaw thrust when ventilating the manikins. All participants were instructed to deliver the rescue breaths over one second and to watch thoracic excursions to avoid overdistention of the stomach and excessive increase of the intrathoracic pressure. Feedback devices (Laerdal SkillGuide; Laerdal Medical AS, Stavanger, Norway) allowed real-time notification about chest compression rate, compression depth, chest recoil, and successful ventilation during training. During the practice session and simulation, but not during the assessment of the resuscitation skills before the retraining, directly after training and in the follow-up, all participants received both immediate verbal feedback from the instructors (for example “push harder,” “minimize interruptions”) and real-time visual feedback from the feedback device regarding the quality of CPR because direct feedback has been demonstrated to increase the quality of CPR (13, 14).

After training CPR, the instructors simulated three different emergency scenarios, each requiring three participants to perform an emergency treatment as realistically as possible:

In the first scenario, a child chokes while eating lunch. The initial coughing turns into gasping and becomes weaker until complete respiratory arrest and unconsciousness. In the second scenario, a child is found lifeless in a paddling pool filled with water with his face submerged. In the third scenario, a child falls from a climbing tower and suffers a head laceration as well as a traumatic brain injury, which leads to a loss of consciousness after a few minutes and finally to respiratory arrest.

After each scenario, a structured debriefing took place according to established debriefing methods (15). Each debriefing included four phases: Participants’ reactions, summary and description of the medical problem, analysis and discussion of the treatment and team dynamics by using facilitated reflection and summary of what was learned. In the reaction phase, impressions and emotions were collected, the description phase identified the medical problem. During the analysis, situations that appeared to need improvement were discussed constructively, but actions that went particularly well and were seen as valuable in managing emergency situations were also highlighted. Finally, participants were asked to report what they had gained for themselves for future emergencies.

After the simulations, participants were invited to repeat the resuscitation with an infant manikin that they had already performed at the beginning of the retraining. After 2 weeks, participants were again given the opportunity to participate in the same mini-survey they had participated in prior to the retraining.

### Follow-Up Evaluation

After 6 months, a follow-up examination was performed on a voluntary basis. Participants were again asked to perform single-rescuer BLS for 2 min. CPR performance was recorded and documented *via* the software in the same way as in the previous assessments.

### Data Collection and Statistical Analysis

The details of the performed CPR steps and intervals were documented by one of the instructors (check for responsiveness, shout for nearby help, look for respirations and check pulse, activate emergency response system) and by a laptop software which analyzed chest compressions and ventilations (Laerdal Wireless SkillReporting, Laerdal Medical AS, Stavanger, Norway). During the CPR rounds the participants did not receive any verbal or visual feedback regarding the quality of chest compressions and rescue breaths. Collected data of the participants and their CPR performance comprised time of last BLS course, chest compression rate, compression depth, chest recoil, correct hand position, successful application of rescue breaths, and duration of no-flow time due to interruptions of chest compression for ventilation. Based on the guidelines, target ranges were set for chest compression rate from 100 to 120/min, for compression depth from 1.5 to 2 inches (4–5 cm), and a compressions-to-breaths ratio of 30:2 (11). Chest recoil was defined as full and correct if no residual thoracic pressure was recorded by the software between compressions. The interruptions of chest compressions for ventilation were expected to be lasting less than 10 s to achieve at least 60 chest compressions per minute at a rate of 100/min (16, 17). A rescue breath was defined as success when it was delivered slowly and resulted in an adequate thoracic excursion. Number of compressions, duration of compression rounds, compression depth and chest recoil, duration of interruption of chest compressions for ventilation, and applied rescue breath volume were recorded by the laptop software and then extracted from the software by the instructors. To allow comparability of the results, a resuscitation score was generated based on the individual components of a guideline-compliant resuscitation. A maximum of seven points could be achieved. A maximum of one point each for number of chest compressions, chest compression rate, compression depth, chest recoil, number of rescue breaths, successful application of MMV, and duration of no-flow time. For example, if 90% of chest compressions had correct compression depths, the participant received 0.9 points.

Statistical analysis and the creation of charts were performed using SigmaPlot (Version 13 for Windows, Systat Software, Inc., San Jose, CA, United States). Normality was assessed using the Shapiro–Wilk test. Data are presented as median [interquartile range (IQR)]. For statistical analysis Student’s *t*-test and the Mann-Whitney Rank Sum test was applied, depending on whether the data were normally distributed. Categorical variables were compared using Two-tailed Fisher’s exact test. In the figures box-and-whisker plots are shown. A probability of *p* < 0.05 was defined as statistically significant.

## Results

In 2019, 39 caregivers of a daycare center participated in the evaluation of our multimodal resuscitation program. 64.1% of participants responded that they last attended a first aid course was two or more years ago. 20.5% last attended within the last 12–24 months. 15.4% did not provide any information on this. A total of 101 simulated cardiopulmonary resuscitations were evaluated.

Table 1 provides an overview of the actions required by the guidelines for a lifeless child before resuscitation efforts are initiated. After retraining and in follow-up, there was a significant improvement of checking for consciousness and calling for help compared to baseline findings. The increase in looking for breathing and making an emergency call failed to achieve statistical significance.

**TABLE 1:** Checking for responsiveness, shouting for nearby help, looking for breathing, and activating the emergency response system before retraining, immediately after training, and in the follow-up.

	Pre-retraining (*n* = 38)	After training (*n* = 39)	Follow up (*n* = 24)
Check for responsiveness (%, time [IQR])	31.6% (2.0 s. [1.0 – 3.8])	94.9% (1.0 s. [1.0 – 2.0]) *p* < 0.001	87.5% (1.0 s. [1.0 – 2.0]) *p* = 0.001
Shout for nearby help (%, time [IQR])	65.8% (7.0 s. [2.5 – 10.0]	61.5% (11.5 s. [7.0 – 16.5]) *p* = 0.81	100.0% (8.0 s. [5.0 – 30.0]) *p* = 0.001
Look for breathing (%, time [IQR])	78.9% (6.0 s. [2.5 – 13.5]	89.7% (7.0 s. [5.0 – 9.0]) *p* = 0.22	95.8% (5.0 s. [4.0 – 8.0]) *p* = 0.14
Activate emergency response system (%, time [IQR])	71.1% (15.0 s. [8.0 – 40.0]	61.5% (60.0 s. [13.0 – 88.3]) *p* = 0.47	91.7% (35.5 s. [12.3 – 78.8]) *p* = 0.06

**FIGURE 1:**
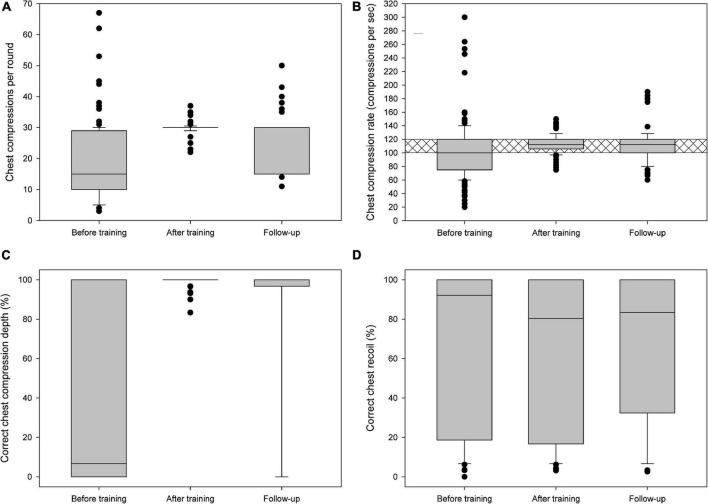
**(A)** Chest compressions per round, **(B)** chest compression rate per second, **(C)** correct chest compression depth, and **(D)** correct chest recoil before training, immediately after training, and in the follow-up. Crosshatching is for guideline recommendations.

**FIGURE 2:**
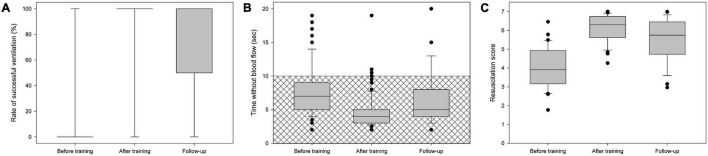
**(A)** Successful rescue breaths, **(B)** duration of rescue breaths (“no-flow time”), and **(C)** resuscitation score before training, immediately after training, and in the follow-up. Crosshatching is for guideline recommendations.

Figures 1, 2 and Table 2, summarize the quality of chest compressions and rescue breaths before retraining, after training, and at follow-up after 6 months. Except for chest recoil, participants correctly performed the other components of the BLS measures (compressions per round, compression rate, correct compression depth, rescue breaths and no flow time) immediately after retraining according to the guidelines and improved them significantly compared to the performance before retraining. Despite retraining, participants showed a tendency to worsen in correct chest recoil, although not statistically significant. The overall resuscitation score was significantly increased.

**TABLE 2:** Chest compressions per round, chest compression rate per second, correct chest compression depth, correct chest recoil, successful rescue breaths, duration of rescue breaths (“no-flow time”), and resuscitation score before retraining, immediately after training, and in the follow-up.

	Before retraining (*n* = 38)	After training (*n* = 39)	Follow up (*n* = 24)
Chest compressions per round (n [IQR])	15.0 [10.0 – 29.0]	30.0 [30.0 – 30.0] *p* < 0.001	30.0 [15.0 – 30.0] *p* < 0.001*
Number of participants with correct amount of chest compressions per round (n, %)	11 (28.9%)	39 (100.0%) *p* < 0.001	15 (62.5%) *p* = 0.02
Chest compression rate (per sec. [IQR])	100.0 [75.0 – 120.0]	112.5 [105.9 – 120.0] *p* < 0.001	112.5 [100.0 – 120.0] *p* = 0.002*
Participants with correct chest compression rate (n, %)	11 (28.9%)	25 (64.1%) *p* = 0.003	11 (45.8%) *p* = 0.28
Correct compression depth (% [IQR])	6.7% [0.0 – 100.0]	100.0% [100.0–100.0] *p* < 0.001	100.0% [96.7–100.0] *p* < 0.001*
Participants with at least 95% correct chest compression depth (n,%)	7 (18.4%)	33 (84.6%) *p* < 0.001	18 (75.0%) *p* < 0.001
Correct chest recoil (% [IQR])	92.1% [18.7 – 100.0]	80.3% [16.7 – 100.0] *p* = 0.10	83.3% [32.4 – 100.0] *p* = 0.48*
Participants with at least 95% correct chest recoil (n,%)	16 (42.1%)	7 (17.9%) *p* = 0.03	7 (29.2%) *p* = 0.42
No-flow time (sec. [IQR])	7.0 s. [5.0 – 9.0]	4.0 s. [3.0 – 5.0] *p* < 0.001	5.0 s. [4.0 – 8.0] *p* = 0.004*
Participants with correct no-flow time (n,%)	36 (94.7%)	33 (84.6%) *p* = 0.26	22 (91.7%) *p* = 1.0
Successful rescue breaths (% [IQR])	0.0% [0.0 – 0.0]	100.0% [100.0 – 100.0] *p* < 0.001	100.0% [50.0–100.0] *p* < 0.001*
Participants with at least 95% successful rescue breaths	5 (13.2%)	24 (61.5%) *p* < 0.001	14 (58.3%) *p* < 0.001
Resuscitation score (n [IQR])	3.9 [3.2 – 4.9]	6.3 [5.6 – 6.7] *p* < 0.001	5.7 [4.7 – 6.4] *p* < 0.001*

*Compared to pre-retraining.

In the follow-up after 6 months, some of the parameters showed slight deterioration compared to the performance immediately after training: Chest compressions per round, no-flow time, compression depths, and resuscitation score. There was no significant change in chest compression rate, success of rescue breaths and correct chest recoil. Compared to pre- retraining all parameters except for chest recoil remained significantly improved (Figures 1, 2 and Table 2).

The mini-survey was answered by 34 participants before the retraining, and by 15 participants after the training. Prior to the retraining, 7 caregivers (20.5%) indicated that they would be afraid to perform resuscitation on a child. The reasons given were: Fear of hurting or injuring the child (3 times); Fear of performing resuscitation procedures incorrectly (4 times); Uncertainty in which cases resuscitation procedures are required (2 times); One participant gave no response. After the training, all caregivers answered that they would perform resuscitation. Before the retraining, 13 participants (38.2%) and after the training, 1 participant (6.7%) stated that they were afraid of possible emergencies in the daycare center. When asked if they felt well prepared for potential emergency situations, 11 participants (32.3%) answered no before the retraining. After the training, all caregivers reported to feel well prepared.

## Discussion

Although cardiac arrest in children is a very rare event, every child who receives no or poor resuscitation is one child too many. Immediate and high-quality resuscitation is associated with improvement in otherwise poor survival and neurologic outcome (1–5). An evaluation of the nationwide registry of pediatric OHCA in Japan revealed an incidence of out of hospital cardiac arrests in nursery schools and kindergartens of 0.13 per 100,000 children per year (18). The causes of cardiac arrest of non-medical origins were suffocation, drowning, and severe traumatic brain injury. This study confirmed our choice of three simulated emergencies that frequently occur in daycare centers. In the Japanese evaluation, a large proportion of patients were also found unconscious during naptime (18). These situations require rapid recognition of cardiac arrest and immediate action, which must also be highlighted in BLS courses. With our training concept, we were able to achieve a significant increase in immediate testing of responsiveness from about 30 to 90%.

Another element of our multimodal training program is the use of expert modeling. This allowed all components of high-quality resuscitation to be demonstrated in a precise and standardized manner by the instructors, as there is reason to assume that without demonstration and guidance, laypersons will have difficulty putting BLS guidelines in practice as other studies reported (8, 10). We also wanted to avoid participants having inhibitions about being the first to practice in front of their colleagues and exposing themselves. In addition, there is research that showed that expert modeling is superior to other teaching techniques and can improve performance on complex tasks (19–21).

The core of our multimodal resuscitation program is simulation-based training of three emergency scenarios followed by a structured and guided debriefing. Recently, simulation-based learning has become a standard for clinical teams and is receiving increasing attention in training laypersons (22, 23). Scenario-based training has been reported for family members of newborns, children with seizures, children with diabetes, or children on home mechanical ventilation (24–27). However, simulation-based training comes with challenges: in order to provide all participants with the opportunity to participate in emergency scenarios, the group size must be small. Also, with an increasing number of participants per instructor, the risk of not detecting errors during training of BLS measures also increases as shown in a randomized controlled simulation study that recommends an instructor-to-participant ratio of 1:6 (28). In addition, trainings must be delivered by instructors with good knowledge of current guidelines and profound experience in educational methods (29, 30). This includes special skills in conducting debriefings as well as creating a psychologically safe learning environment (31). Failures in treatment during simulation are common. Due to the realistic nature of the emergency scenarios, the participants also need to be emotionally picked up and guided during the debriefing. There is a considerable risk that participants feel ashamed in front of their colleagues, blame themselves for making mistakes and develop restraints to take part in further training sessions or even to perform first aid measures in real life. The effort in both time and special knowledge of personnel that is required for our training concept is higher compared to conventional BLS training courses for laypersons. Until now our participants received BLS training in large groups of about 20 participants per instructor at intervals of about 2 years, which was conducted by paramedics. For comparison, our training program required 2 certified PALS instructors and 1 assistant who was a certified PALS provider for 7–8 participants. However, the savings of the former approach must be contrasted with the results of our study. The majority of our participants had last attended a conventional BLS course about 2 years ago. Our pre-training assessment revealed that most compressions and rescue breaths were insufficient. This would have resulted in poor performance in real emergency situations. Although our follow-up period did not cover 2 years, our cohort still showed good adherence to the current guidelines and significantly better resuscitation performance after 6 months than in the pre-retraining assessment. Provocatively speaking, poor training is hardly better than no training at all. Adequate training of laypersons in BLS measures certainly requires the investment of enough time and resources. There is another argument why it makes sense to train BLS measures thoroughly, even if the probability of a cardiac arrest in children is very low: the trained caregivers can also react adequately in the much more frequent adult emergencies. To better serve this aspect, it would be possible to additionally address the differences from adult resuscitation in our pediatric BLS training program.

Despite many different existing training formats, there is currently no evidence on the optimal training method for lay rescuers (32). In Germany, about 1 million citizens are trained annually with standardized first aid courses (10). Nevertheless, the bystander CPR rate is below 20% (33). An evaluation of the courses showed that the BLS elements were satisfactorily taught. However, there were significant deficiencies with respect to the realism of the exercises and the reduction of fears of making mistakes or causing harm (10). Therefore, the development of our course concept focused on the implementation of realistic scenarios to better port the BLS measures into practice and to be able to identify and reduce fears of mistakes in the debriefing. In addition to basic resuscitation skills, local logistical challenges such as making an emergency call, dealing with other children, and briefing paramedic personnel were also integrated into the training. Therefore, the training was conducted on-site. Scenario-based course concepts are available from both the AHA and ERC, but are designed for health care professionals (e.g., AHA BLS or PALS course). For laypersons, there is a First Aid CPR AED course from the AHA, but it only includes infant and child CPR as an optional module and no simulated scenarios that were offered the participants of our training program. Due to the lack of existing simulation-based course concepts for lay persons, the purpose was to develop a training course with BLS content and scenarios tailored to the specifics of the daycare center. The subsequent written evaluation showed that participants’ fears were reduced and that they would feel confident to perform resuscitation on a child. Only one person was anxious about potential emergencies at work compared to 13 of 21 caregivers before attending our training program.

The optimal timing of retraining is not known. Most resuscitation courses rely on an interval of every 1–2 years (32). Studies have shown that resuscitation skills decline after only a few months (34–36). In our study, resuscitation score, chest compressions per round and compression depths were already significantly worse after 6 months than immediately after training. Regular training in intervals of 1–6 months was associated with improved resuscitation skills (32, 36, 37). Thus, significantly shorter training intervals than 2 years seem desirable. However until now, there is no evidence about the optimal training method, group size and refreshing interval.

Besides the limitation that our study covered only a 6-month follow-up period, it should be noted that only 24 of the 39 participants could be reached for a follow-up. Therefore, we cannot say with certainty whether this biased the results. In addition, the evaluation of correct chest recoil showed no improvement compared to the values before the training. Although the importance of a full chest recoil during the training was highlighted and had been demonstrated accordingly, this could apparently not be translated into practice by the participants. It is remarkable that the participants were able to reach almost perfect compression depth during chest compressions and rescue breaths directly after the training and in the follow-up examination. These results are better than results of other studies (38, 39). As a limitation, it must be mentioned that pre-training compression depth and rescue breaths were performed correctly only with a median of 6.7 and 0.0%, respectively. Therefore, there was a strong focus on demonstration and practice of the correct compression depth and rescue breaths. In addition, the functionality of the manikins cannot be changed and the ventilation always follows the same mechanism. If a participant masters the correct ventilation technique once, then he or she is very likely to do this consistently from that point on. This does not apply to reality and is a general limitation of simulation studies. In contrast, the rate of full chest recoil was not improved, but tended to worsen, although not statistically significantly. Obviously, we have been able to communicate the performance of correct compression depth and rescue breaths much better than the importance of full chest recoil. Based on this experience, better attention needs to be paid to more balanced training of the different BLS measures in future trainings. Another limitation of our study is the response rate to the mini-survey of 38.5% after training. The participants who did not respond may have a different opinion of our multimodal resuscitation course concept than those who responded.

## Conclusion

Our multimodal cardiopulmonary resuscitation training for caregivers resulted in significant improvement of resuscitation skills of the participants. Although the effect diminished within 6 months, adherence to the resuscitation guidelines was significantly better than before the training. The considerable investment of time and personnel seems to pay off in terms of resuscitation skills compared to conventional training in large groups. Future research is needed to identify optimal training method, group size and interval.

## Data Availability Statement

The raw data supporting the conclusions of this article will be made available by the authors, without undue reservation.

## Ethics Statement

The studies involving human participants were reviewed and approved by the Ethics Committee of the University Hospital Tübingen, Germany. Written informed consent for participation was not required for this study in accordance with the national legislation and the institutional requirements.

## Author Contributions

JM, EH, and FN designed the study. JM, TI, and EH contributed to the data collection, data analysis, and data interpretation. JM wrote the first draft of the manuscript. All authors contributed to the article and approved the submitted version.

## Conflict of Interest

The authors declare that the research was conducted in the absence of any commercial or financial relationships that could be construed as a potential conflict of interest.

## Publisher’s Note

All claims expressed in this article are solely those of the authors and do not necessarily represent those of their affiliated organizations, or those of the publisher, the editors and the reviewers. Any product that may be evaluated in this article, or claim that may be made by its manufacturer, is not guaranteed or endorsed by the publisher.
